# Short-term starvation synergistically enhances cytotoxicity of Niraparib via Akt/mTOR signaling pathway in ovarian cancer therapy

**DOI:** 10.1186/s12935-022-02447-8

**Published:** 2022-01-11

**Authors:** Wang Zhi, Suting Li, Yuting Wan, Fuwen Wu, Li Hong

**Affiliations:** Department of Gynecology and Obstetrics, Renmin Hospital of Wuhan University, 238 Jiefang Road, Wuhan, Hubei Province People’s Republic of China

**Keywords:** Niraparib, Short-term starvation (STS), DNA damage, Akt/mTOR

## Abstract

**Background:**

Short-term starvation (STS) has gradually been confirmed as a treatment method that synergistically enhances the effect of chemotherapy on malignant tumours. In clinical applications, there are still some limitations of poly (ADP-ribose) polymerase inhibitors (PARPi), including understanding their effectiveness and side effects. Here, we sought to investigate the effect and mechanism of the combined use of STS and niraparib in the treatment of ovarian cancer.

**Methods:**

In in vitro experiments, SKOV3 and A2780 ovarian cancer cells were treated with STS and niraparib alone or in combination. Cell viability was assessed with CCK-8, and cell cycle, apoptosis, DNA damage repair and autophagy were examined to explore the molecular mechanisms. Akt and mTOR inhibitors were used to examine any changes in DNA damage repair levels. Xenograft animal models were treated with STS and niraparib, and HE staining and immunohistochemistry were performed to examine the effects.

**Results:**

The combined use of STS and niraparib inhibited cell proliferation and increased apoptosis more than niraparib application alone. In addition, compared with the niraparib group, the STS + niraparib group had increased G2/M arrest, DNA damage and autophagy, which indicated that STS pretreatment enhanced the cytotoxicity of niraparib. In animal experiments, STS did not affect the growth of transplanted tumours, but the combined treatment synergistically enhanced the cytotoxicity of niraparib. In in vivo experiments, STS did not affect the growth of transplanted tumours, but the combined treatment synergistically enhanced the cytotoxicity of niraparib and reduced the small intestinal side effects caused by niraparib chemotherapy.

**Conclusion:**

STS pretreatment can synergistically enhance the cytotoxicity of niraparib. STS + niraparib is a potentially effective strategy in the maintenance therapy of ovarian cancer.

## Introduction

Epithelial ovarian cancer (EOC), as the most lethal gynaecological malignancy, shows a higher mortality rate than other common gynaecological malignancies, such as endometrial cancer [1]. Extensive cytoreductive surgery and platinum-based chemotherapy are routine upfront treatments [2]. Early-stage ovarian cancer is highly curable, but 70% of women present with stage III/IV disease, resulting in a low overall survival rate of ~ 40% [3]. Extending the survival period and improving the quality of life of patients are the main goals of current EOC treatment.

Poly (ADP-ribose) polymerase inhibitors (PARPi) are expected to be an effective drug for the treatment of ovarian cancer by targeting DNA repair. PARP-1 is involved in DNA repair through different pathways, such as homologous recombination (HR), nucleotide excision repair (NER), alternative nonhomologous end-joining (alt-NHEJ) and single-strand DNA break (SSB) repair [4]. PARPi induces PARP DNA entrapment and destabilizes replication forks, exacerbating replication deficiencies and motor catastrophe [5]. Niraparib is an approved oral PARPi for the maintenance treatment of ovarian cancer. PARPi was initially used to treat ovarian cancer patients with BRCA1 mutations. Regardless of the occurrence of BRCA mutations, the effectiveness of PARPi for the maintenance treatment of ovarian cancer has been verified in many clinical studies [6, 7]. Nevertheless, a large number of patients still fail to benefit from PARPi, and drug resistance is one of the main reasons [8–10]. Overcoming drug resistance is one of the keys to maintenance treatment of ovarian cancer, and combination strategies are a scientifically rational way to target alternative DNA repair pathways to improve PARPi sensitivity. Some research has already explored strategies of PARPi combined with VEGFR inhibitors [11], PI3K inhibitors [12], CDK inhibitors [13], PD-L1 blockade [14] and so on, and some of them have entered clinical trials.

The effectiveness of short-term starvation (STS) or calorie restriction combined with chemotherapy in the treatment of malignant tumours has been widely demonstrated. Studies have shown that STS selectively protects normal cells while making malignant cells more sensitive to chemotherapy, which is called differential stress resistance [15, 16]. In addition, STS can synergistically reduce the side effects of chemotherapy, making STS more attractive [17]. However, the effect of STS on the maintenance treatment of ovarian cancer and the efficacy of PARPis have not yet been elucidated. This study evaluated the preclinical efficacy of STS in combination with niraparib in the chemotherapy of BRCA1 wild-type ovarian cancer cells. We performed in vivo and in vitro experiments and showed that STS synergistically enhanced the cytotoxicity of niraparib, including intensive double-strand DNA damage repair, inhibition of cell proliferation, apoptosis induction, cell cycle arrest and enhanced autophagic flux, and Akt/mTOR signalling was involved in this process [18]. Our research indicates that STS combined with the PARPi niraparib is a promising treatment of EOC.

## Material and methods

### Cell culture and reagents

SKOV3 and A2780 cell lines were obtained from the China Center for Type Culture Collection (CCTCC, Wuhan, China) and cultured in RPMI-1640 (GIBCO-BRL, Gaithersburg, MD, USA) with 10% fetal bovine serum (FBS, Yeasen, Shanghai, China) and 1% antibiotics (penicillin and streptomycin, Yeasen, Shanghai, China) in an incubator with 5% CO_2_ at 37 °C. Niraparib was obtained from Zai Lab Co.,Ltd (Suzhou, China), and dissolved in dimethyl sulfoxide (DMSO, Yeasen, 60313ES60). The glucose content in the cell culture medium of different groups is dissimilar. Compared with the control culture medium with 2.0 g/L glucose and 10% FBS, STS group was performed in RPMI-1640 medium with 0.5 g/L glucose and 1% FBS for 24 h and then incubated back to control medium for niraparib or other follow-up experiments. Other reagents sources were listed below: trypsin/EDTA solution (HyClone, Utah, USA), Cell Counting Kit (CCK)-8 (Multiscience Biotech, China), MK-2206 (MCE, HY10358) and AZD8055 (MCE, HY-10422).

### Clonogenic assay and cell viability analysis

SKOV3 and A2780 cells were seeded to 60–70% confluency and treated for indicated time. After treatment, cells were digested and plated evenly on 6-well plates at density of 500 cells/well. After growing 8–14 days for colony formation, cells were washed with PBS, fixed with methylalcohol for 20 min and stained with 0.5% crystal violet for 20 min. The number of colonies was quantified by Image J software in three independent experiments.

PerkinElmer Victor3 1420 Multilabel Counter (Waltham, MA) was used to performed the cell viability assay. Cell Counting Kit (CCK)-8 was obtained from Multiscience Biotech, China. Cells were plated with a density of 5000 cells/well into 96-well plates and then incubated with different concentration of niraparib (0, 1.25, 2.5, 5, 10, 20, 40 μM) for 24 or 48 h. 10μL CCK-8 was added into each well and cells were incubated in incubator with 5% CO_2_ at 37 °C for 2 h. All assays were conducted in triplicate. The absorbance of each group of cells was detected at 450 nm wavelength.

### Western blot

Cells were treated with RIPA Lysis Buffer, and protein concentrations were quantitated by BCA assay kit (P0013B, Beyotime, Shanghai, China). Primary antibodies used were: anti-PARP-1 (13371-1-AP, Proteintech, 1:1000), anti-cleaved caspase3(#9664, CST, 1:1000), anti-GAPDH (10494-1-AP, Proteintech, 1:5000), anti-Bcl2 (12789-1-AP, Proteintech,1:1000), anti-Bax (50599-2-Ig), anti-γH2AX (ab229914, abcam, 1:2000), anti-RAD51 (ab133534, abcam,1:2000), anti-phospho-Akt (phospho S473) (ab18622, abcam, 1:500),anti-Akt (10716-2-AP, Proteintech,1:1000), anti-phospho-mTOR (phospho S2448) (abcam,ab109268, 1:3000), anti-mTOR (abcam,ab134903, 1:10,000), anti-p62 (MBL, M162-3, 1:5000), anti-LC3I/II (14600-1-AP, Proteintech, 1:1000). Secondary antibodies used were: HRP goat anti-rabbit IgG (BL003A, Biosharp, 1:5000), HRP goat anti-mouse IgG (BL001A, Biosharp, 1:5000).

Samples of whole-cell lysate containing equal amounts of protein was loaded on 10% SDS-PAGE and then transfer to PVDF membranes (0.45 μm, EMD Millipore, Billerica, MA, USA). After being blocked in TBST with 5% non-fat milk, the PVDF membranes were incubated with primary antibody overnight at 4 °C and then with horseradish peroxidase (HRP)-conjugated secondary for 1 h. PVDF membranes were visualized using a chemiluminescence substrate kit (Pierce™ ECL Western Blotting Substrate; Thermo Scientific Fisher, Inc.). Immunoblots were quantitated using Image J (v1.8.0).

### Autophagy flux analysis

mRFP-GFP-LC3 adenoviral (HanBio Technology, Shanghai, China) was used to transfect SKOV3 and A2780 cells. Then, the cells were incubated in control medium containing 5 μM niraparib or STS medium for indicated time. Autophagy flux assay were performed with a laser scanning confocal microscopy (FV1200, Olympus Corp, Tokyo, Japan).

### Flow cytometry for apoptosis and cell cycle analysis

For apoptosis analysis, cells were suspended with 100 μl of 1 × binding buffer and stained with Annexin V-PE and 7-AAD (Annexin V-PE Apoptosis Detection kit; BD Pharmingen; SanDiego, CA, USA) for 15 min at 37 °C and 400 μl binding buffer was added. For cell cycle analysis, cells were harvested, centrifuged and added into 70% ice-cold ethanol and incubated at 4 °C overnight. In the second day, cells were incubated in cell cycle staining solution (Biosharp, BL105A) with 20 μg/ml propidium iodide, 200 μg/ml RNAase A and 0.1% Triton X-100 for 1 h. Apoptosis and cell cycle analysis were carried by BD FACSAria (BD Biosciences, Franklin Lakes, NJ, USA). Data were visualized using Flowjo Software (Flowjo 10.6.2, LLC, Ashland, OR, USA). All assays were conducted in triplicate.

### Immunofluorescence staining

SKOV3 and A2780 cells grown on glass coverslips and were fixed in 4% formaldehyde for 10 min and then permeabilized for 15 min in 1% Triton X-100. After blocked with 5% bovine serum albumin for 1 h at room temperature, cells were incubated in primary antibodies including γH2AX (1:100) and RAD51 (1:500) overnight at 4 °C and then in Alexa 488-conjuated secondary antibody (1:500, Thermo Fisher Scientific, A-11070) for 1 h at room temperature. Glass coverslips were mounted in anti-fade mounting medium containing DAPI (Biosharp, BL739A) and analyzed under laser scanning confocal microscopy (FV1200, Olympus Corp, Tokyo, Japan).

### Comet assay

Comet assay were performed under alkaline conditions using the Comet Assay Kit (Abcam, ab238544) according to the manufacturer’s instructions. Briefly, cells were treated with STS for 24 h or 5 μM niraparib or their combination, harvested and blended with 0.5% low melting point agarose at a ratio of 1:10 (volume/volume). Spread the mixture evenly on a slide and immersed in lysis solution for 20 min. After that, slides were electrophoresis in a horizontal electrophoresis apparatus under conditions of 25 V, 300 mA and then stained with Acridine orange to visualize cellular DNA. The image acquisition was performed with orthographic microscope (BX53F2, Olympus, Tokyo, Japan). All assays were conducted in triplicate. Comet assay software project (CASP) was used to analysis the Tail Moment of each comet, which represents the level of DNA damage.

### ATP assay

According to the manufacturer’s instructions, ATP assay Kit (S20026, Beyotime, Shanghai, China) was used to measure the intracellular ATP level. Cells were treatment as mentioned above in this study. Lysis cell homogenate were centrifuged at 12,000 rpm at 4 °C for 10 min and 100 μl ATP detection reagent was mixed with 20 μl supernatant in a 96-well plate and the luminescence (RLU) of each group was detected by PerkinElmer Victor3 1420 Multilabel Counter (Waltham, MA).

### Tumor xenograft study

All animal experiments were conducted in compliance with the National Institute of Health guidelines for animal research and approved by the Institutional Animal Care and Use Committee of Renmin Hospital of Wuhan University. 6 weeks old female BALB/c nude mice (nu/nu) were purchased from the Beijing Vital River Laboratory Animal Technology Cooperation (Beijing, China). Logarithmic growth phase A2780 tumor cells (1 × 10^7^) were subcutaneous injections into female mice aged 7–8 weeks under the skin of neck and back. When the volume of tumor were approximately 50mm^3^ (day 17), the mice were randomly grouped and used in subsequent experiments (n = 7). niraparib group was given niraparib intragastric injections (50 mg/kg) once a day and 5 days a week. STS group fasted for 48 h per week. The STS + Niraparib group was given a short-term starvation for 2 days before the intragastric injection of niraparib for 5 days per week. After three weeks treatment, nude mice were anaesthetized with isoflurane and sacrificed by the cervical dislocation method, important organ and tumor tissues were collected for further analysis.

### Histopathology and immunohistochemistry

Briefly, tissues were fixed with 10% formalin and embedded in paraffin, 4 μm paraffin embedded were performed and stained with H&E or IHC. The primary antibodies used were Ki67 (27309–1-AP, Proteintech, 1:200), γH2AX (1:100) and RAD51 (1:500). Major organ sections and primary tumors were performed with H&E staining. Images were collected using Microscope (BX53F2, Olympus, Tokyo, Japan) and IHC stained images were analyzed by Image J 1.8.0.

### Statistical analysis

Data were means ± standard deviation of three independent experiments or 7 independent samples. GraphPad Prism software version 7.0 (San Diego CA, USA) were used to perform statistical analysis. One-way analysis of variance (ANOVA) was used for multiple comparisons in three or more groups and unpaired t-tests were applied to determine significance for comparisons in two groups. *P* values < 0.05 were considered significant statistically.

## Results

### Niraparib reduces PARP-1 expression and inhibits SKOV3 and A2780 cell proliferation

**Fig. 1:**
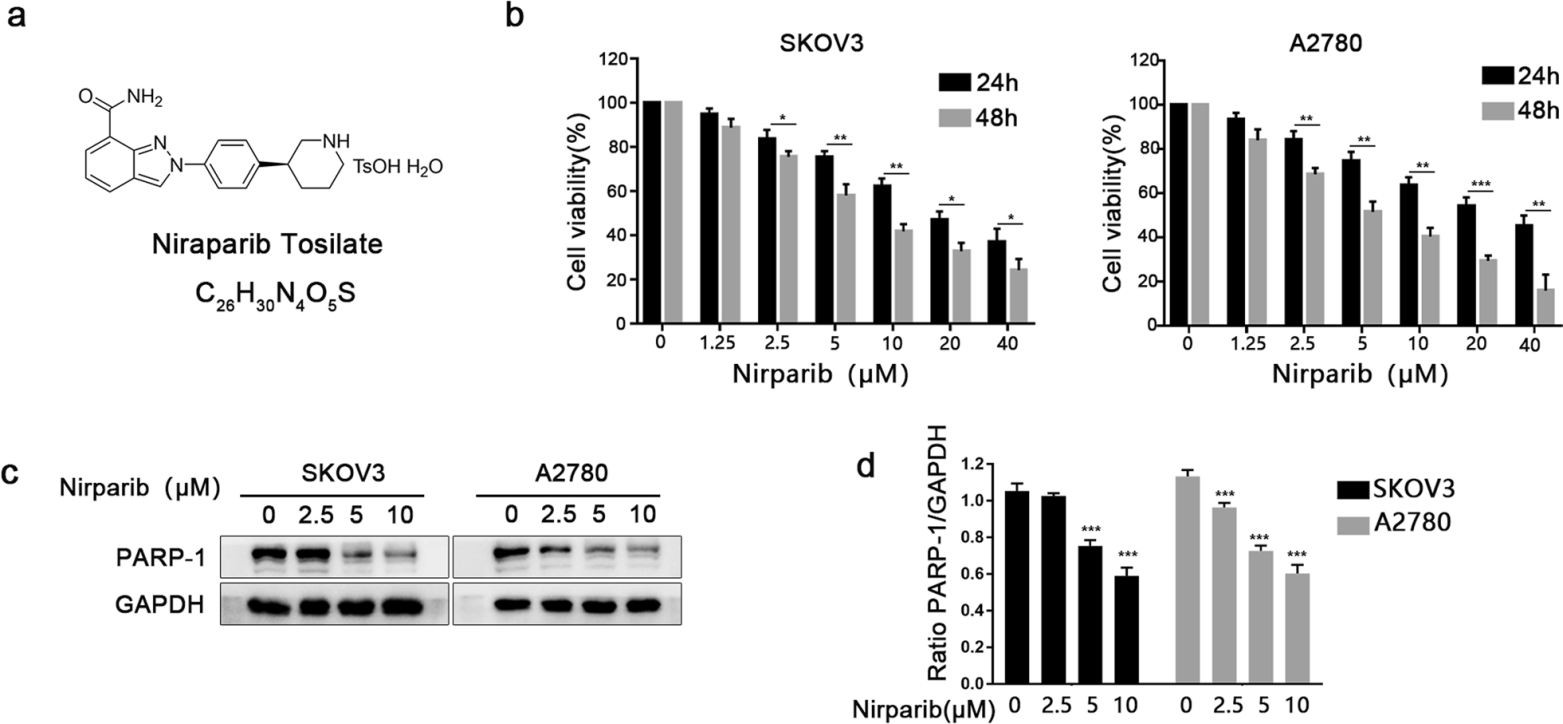
**a** The molecular structure of niraparib. **b** Cells were incubated with niraparib (0, 2.5, 5, 10, 20, 40 μM) for 24 and 48 h, and the proliferation rate was determined by the CCK-8 assay. **c**, **d** SKOV3 and A2780 cells were treated with niraparib at various concentrations (0, 2.5, 5, 10 μM) for 48 h, and the expression of PARP-1 protein was detected by western blot analysis. GAPDH protein was used as a loading control. GAPDH protein was used as a loading control. Data represent the mean ± SD of three independent experiments. ***P < 0.001, **P < 0.01, *P < 0.05

The chemical structure of niraparib is displayed in Fig. 1a. For the CCK-8 assay, SKOV3 and A2780 cells were incubated in 0–40 μM niraparib for 24 or 48 h (Fig. 1b), and the results indicated that after niraparib treatment, the proliferation of SKOV3 and A2780 cells decreased significantly in dose- and time-dependent manners. Next, both SKOV3 and A2780 cells were incubated in 0 μM, 2.5 μM, 5 μM, and 10 μM niraparib for 48 h, and western blotting was performed to analyse the protein expression of PARP-1. Niraparib suppressed the expression of PARP-1 in a dose-dependent manner in both cell lines, especially at concentrations of 5 μM and 10 μM (Fig. 1c and d). In summary, 5 μM niraparib was sufficient to block cell proliferation and reduce PARP-1 expression and could be used for subsequent experimental treatment.

### STS enhances the niraparib chemosensitivity of SKOV3 and A2780 cells

**Fig. 2:**
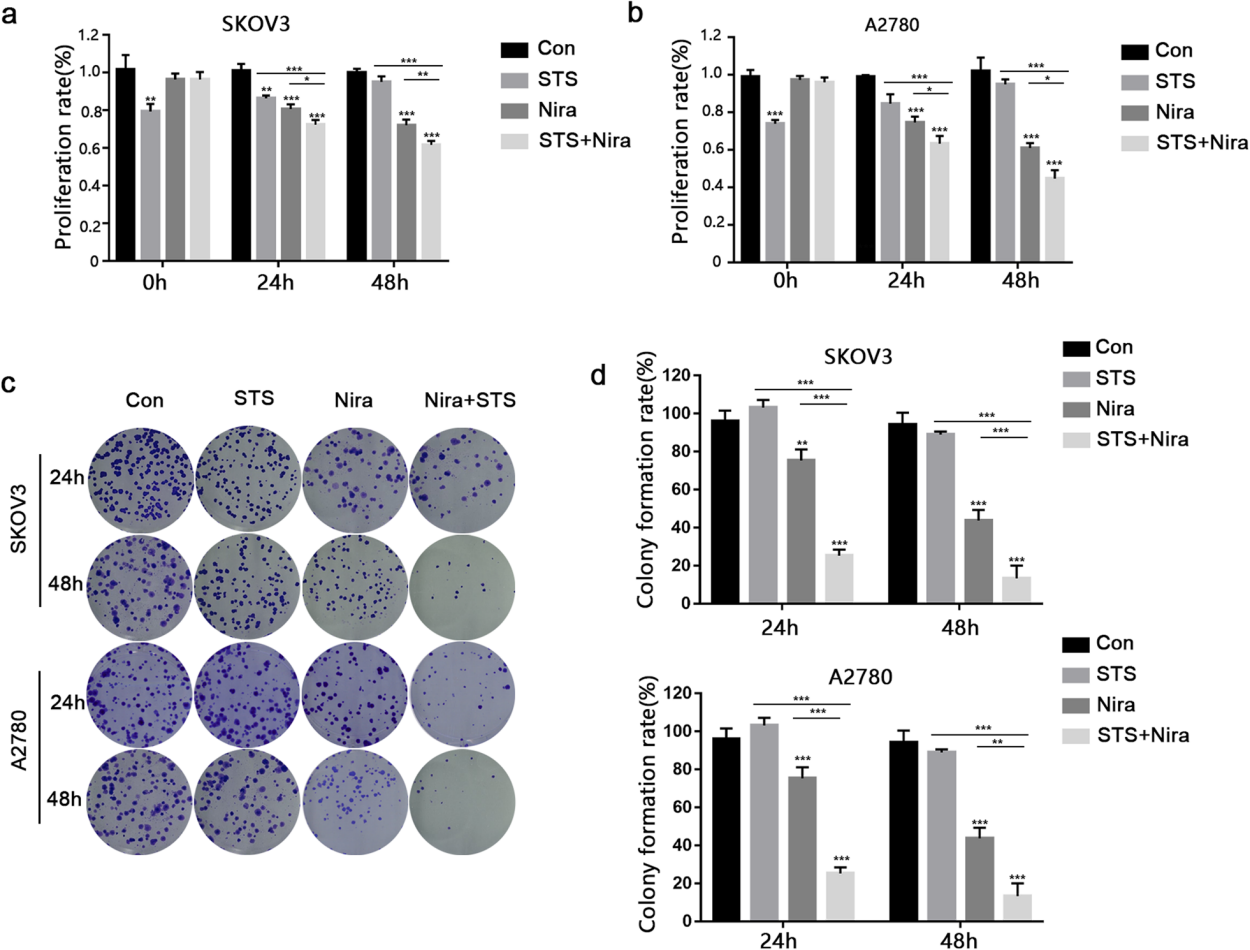
SKOV3 and A2780 cells were treated with STS, 5 μM niraparib or both for 0, 24 and 48 h (time incubated for niraparib) and then detected with the CCK-8 assay. **c**–**e** Twenty-four hours or 48 h after niraparib treatment, a colony formation assay was performed with control medium (10% FBS and 2.0 g/L glucose) for 10 days. Representative images are shown. Three independent experiments were performed. ***P < 0.001, **P < 0.01, *P < 0.05

A CCK-8 assay was performed to clarify the role of niraparib in cells grown in control medium (CM) or STS medium. Niraparib (5 μM) was applied for 24 and 48 h. Compared with the STS and niraparib groups, the combination of niraparib and STS (STS + niraparib) induced a significant inhibition of cell viability for both SKOV3 and A2780 cells after 48 h of treatment. In addition, STS had no obvious effect on cell proliferation after 48 h (Fig. 2a and b).

To better illustrate the role of niraparib in ovarian cancer cells, we performed a colony formation assay. Our results showed that in both SKOV3 and A2780 cells, STS + niraparib had a stronger inhibitory effect on cell proliferation than niraparib alone, and the long-term effect on cell proliferation of the STS group was not obvious (Fig. 2c and d). Collectively, STS enhances niraparib's inhibitory effect on cell proliferation in SKOV3 and A2780 cells.

### STS synergistically enhances apoptosis and G2/M phase arrest induced by niraparib

**Fig. 3:**
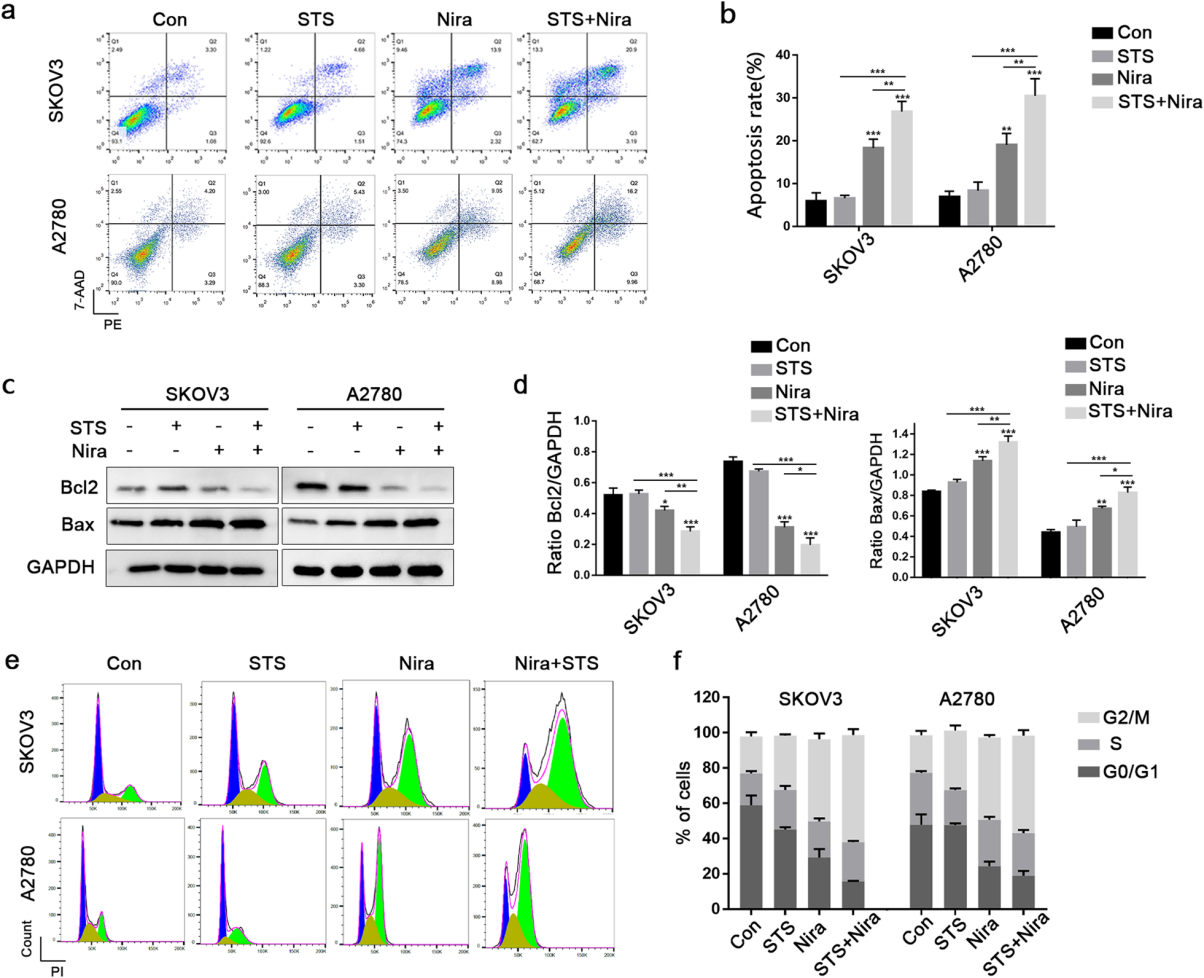
SKOV3 and A2780 cells were treated with niraparib and STS alone or in combination. Niraparib was applied for 48 h. **a**, **b** Apoptosis was detected with an Annexin V-PE/7-AAD kit and analysed by flow cytometry. **c**, **d** The protein expression of Bcl2 and Bax was detected by western blot. GAPDH protein was used as a loading control. **e**, **f** Cell cycle analyses were conducted by flow cytometry, and statistical quantification of the percentages of cells in the different cell cycles is presented. Three independent experiments were performed. ***P < 0.001, **P < 0.01, *P < 0.05

Next, we assessed the effects of the niraparib and STS combination on the apoptosis of ovarian cancer cells. Annexin V-PE/7-AAD double staining was performed by flow cytometry analysis. Compared with the control group, STS had no significant effect on apoptosis, while obvious apoptosis was observed after 5 μM niraparib treatment for 48 h. In addition, STS + niraparib caused an approximately 10% increase in the apoptosis rate in both SKOV3 and A2780 cells compared with niraparib application alone (Fig. 3a and b). Furthermore, western blotting was used to evaluate apoptosis signals. Compared with the niraparib group, the expression of Bax in the STS + niraparib group was significantly increased, and Bcl2 was decreased. Compared with the control, no significant difference was observed in Bax and Bcl2 expression in the STS group (Fig. 3c and d).

We further analysed the effect of niraparib and STS on cell cycle arrest. In SKOV3 and A2780 cells, the proportion of cells in the G2/M period increased after niraparib treatment, and STS had no notable effect on cell cycle arrest. In addition, after STS + niraparib combination application, the proportion of cells in G2/M phase increased more than that in the niraparib group (Fig. 3e and f). These results demonstrate that STS enhances the effects of niraparib on cell apoptosis and G2/M phase arrest, and STS is a potential effective adjuvant treatment among ovarian cancer patients receiving niraparib as maintenance therapy.

### STS pretreatment enhances the effects of DNA damage caused by niraparib

**Fig. 4:**
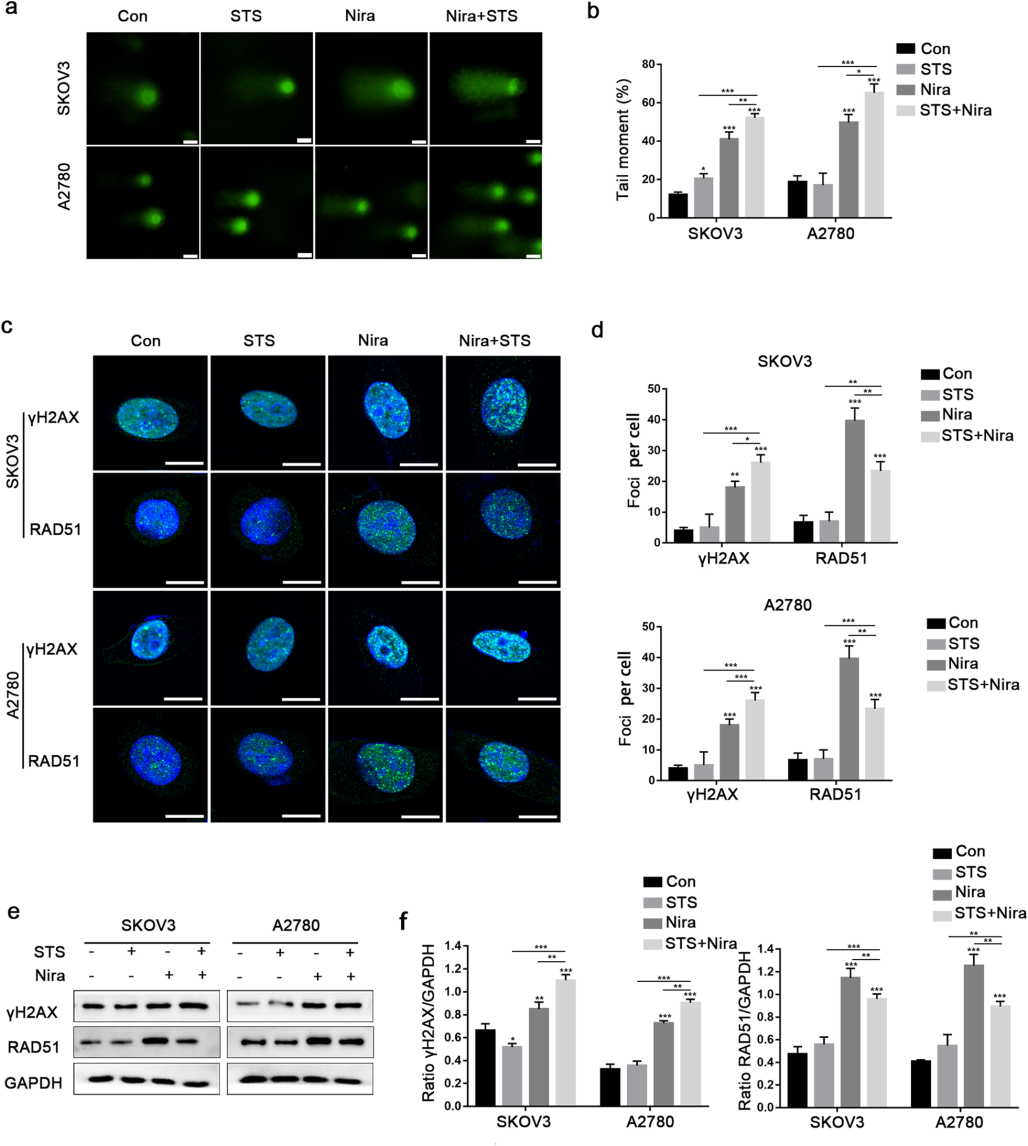
SKOV3 and A2780 cells were treated with niraparib and STS alone or in combination. **a**, **b** Comet assay in SKOV3 and A2780 cells. We quantified the percentage of DNA in the tails to calculate the DNA damage, and each data point represents 50 cells counted. Scale bar, 20 μM. **c**, **d** Immunofluorescence assay in SKOV3 and A2780 cells representing γH2AX and RAD51 foci. Scale bar, 10 μM. **e**, **f** Cells were treated with niraparib, STS or their combination, and western blot analysis was used to detect the expression levels of γH2AX and RAD51. GAPDH protein was used as a loading control. The values are presented as the mean ± SD and were analysed using GraphPad Prism (one-way ANOVA). ***P < 0.001, **P < 0.01, *P < 0.05

Based on the findings in Fig. 3, we reasoned that the enhanced effect of STS on niraparib-mediated ovarian cancer cell apoptosis might be caused by DNA damage. For further verification, an alkaline comet assay was performed to determine the DNA damage associated with STS in SKOV3 and A2780 cells. The amount of DNA damage in the combination therapy group was increased compared to that in the niraparib or STS alone groups (Fig. 4a and b). γH2AX reflects DNA double-strand breaks, and RAD51 is a biomarker of HR during double-strand break repair. Immunofluorescence of γH2AX and RAD51 was performed where the number of γH2AX and RAD51 foci reflects the severity of DNA damage. Consistent with the previous results, STS alone had no effect on γH2AX foci or RAD51 formation, but STS + niraparib resulted in more γH2AX formation and less RAD51 foci accumulation, indicating an increase in the DNA damage level and inhibition of HR repair (Fig. 4c and d). In addition, the western blot results for γH2AX and RAD51 were consistent with the immunofluorescence results (Fig. 4e and f). Overall, these results support the hypothesis that STS may increase the chemotherapy effect of niraparib.

### STS inhibits the Akt/mTOR signalling pathway and enhances autophagic flux

**Fig. 5:**
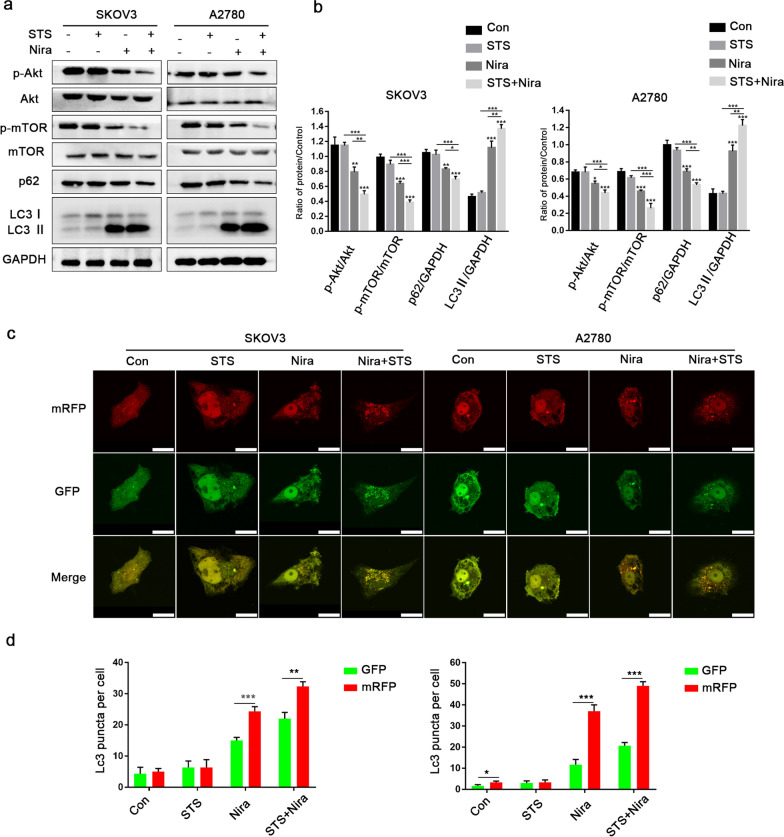
Both SKOV3 and A2780 cells were treated with STS, niraparib or their combination. **a**, **b** After SKOV3 and A2780 cells were transfected with mRFP-GFP-LC3, punctate dots (green and red dots) were detected by laser confocal microscopy. Positive signals were defined if the cell had five or more LC3 puncta in the cytoplasm. Scale bar: 20 μm. **c**, **d** Phosphorylated Akt (p-Akt), p-mTOR, p62 and LC3 were detected by western blot. GAPDH protein was used as a loading control. Three independent experiments were performed. ***P < 0.001, **P < 0.01, *P < 0.05

Previous studies have shown that the Akt/mTOR signalling pathway may be related to PARPi resistance and that STS enhances the effect of chemotherapy through IGF1/PI3K/Akt/mTOR signalling [19–21]. Therefore, we speculated that in this study, STS was enhancing the chemotherapy sensitivity of niraparib by affecting Akt/mTOR signalling. Western blot results showed that in both SKOV3 and A2780 cells, compared with niraparib alone, STS combined with niraparib induced a decrease in the phosphorylation levels of Akt and mTOR, while the expression of total Akt and mTOR did not change significantly. Our findings also showed that STS + niraparib led to the downregulation of p62 and an increase in LC3II (Fig. 5a and b). Moreover, we performed a tandem mRFP-GFP-LC3 adenovirus transfection experiment to clarify the progression of autophagy during the application of STS, niraparib or their combination. For the mRFP-GFP-LC3 vector, GFP was easily degradable in an acidic environment, while the mRFP signal was relatively stable. In the control and STS groups, only slightly accumulated yellow autophagic LC3 (mRFP^+^/GFP^+^) puncta were observed. In the niraparib group, a mass of red puncta of mRFP-LC3 (mRFP + /GFP-) was observed in the cytoplasm, while more red puncta were detected in the STS + niraparib group (Fig. 5c and d). Consistent with the western blot results, niraparib induced the accumulation of autophagosomes and autophagolysosomes. When combined with STS, autophagic flux was further enhanced.

### Inhibition of the Akt/mTOR signalling pathway inhibits DNA damage induced by niraparib combined with STS

**Fig. 6:**
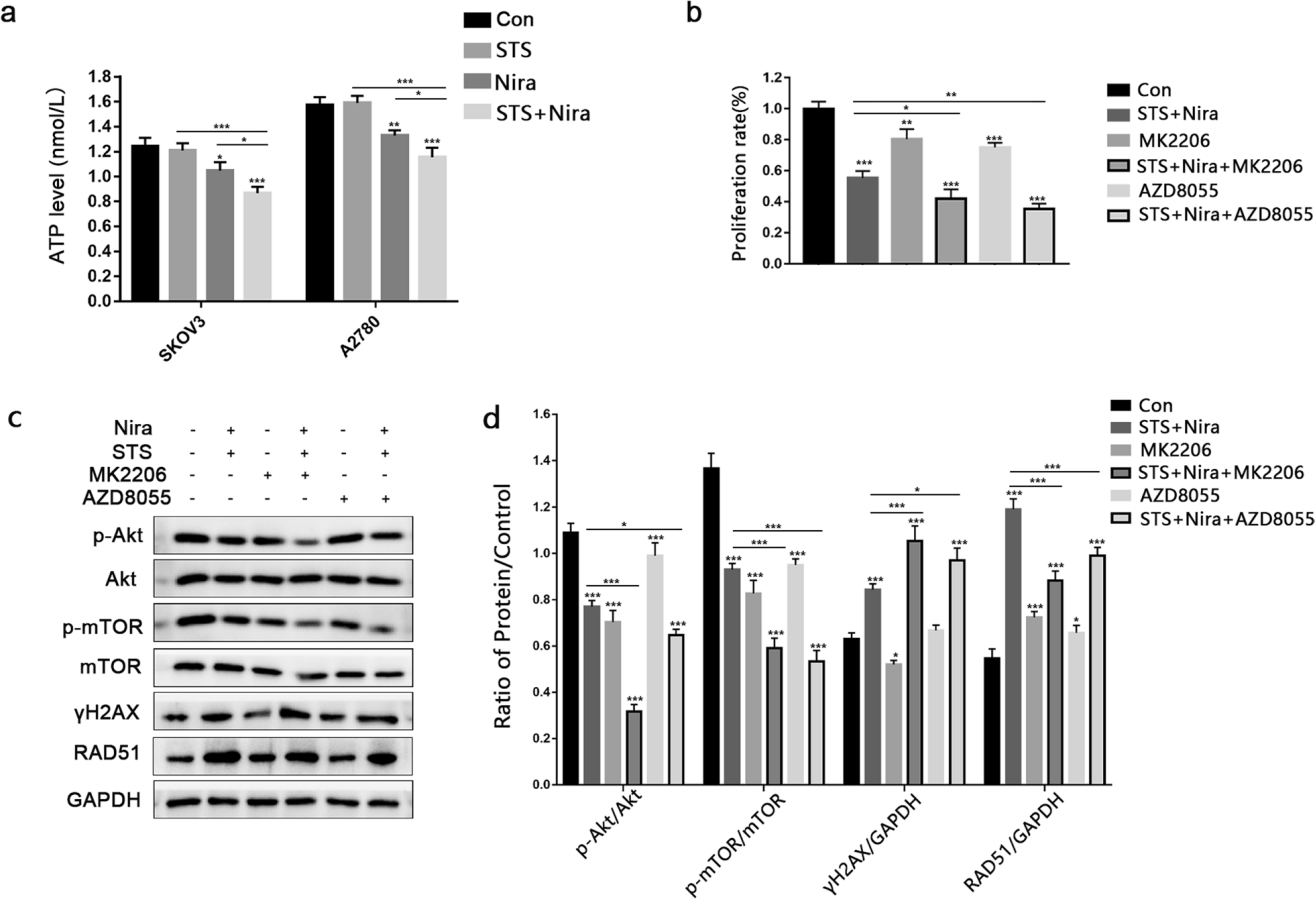
**a** SKOV3 and A2780 cells were treated with STS, niraparib or in combination, and the ATP level was detected by an ATP assay kit. **b** A2780 cells were treated with or without niraparib and STS in combination with MK2206 (300 nM) or AZD8055 (80 nM) for 48 h, and cell viability was determined by a CCK-8 assay. **c** The cells were treated as in Fig. 6b. Phosphorylated Akt (p-Akt), p-mTOR, γH2AX, and RAD51 were detected by western blot. GAPDH protein was used as a loading control. Three independent experiments were performed. ***P < 0.001, **P < 0.01, *P < 0.05

Intracellular energy is directly related to DNA repair. In our study, we observed that the intracellular ATP level in the niraparib group was significantly lower than that in the STS + niraparib group (Fig. 6a). Accumulated evidence has shown that STS enhances the chemotherapy effect of niraparib, such as inhibiting cell proliferation, inducing apoptosis, activating complete autophagic flux and inducing DNA damage, and the AKT/mTOR pathway plays an important role in this process. To further investigate the role of Akt/mTOR, we used MK2206 and AZD8055 to inhibit this signalling pathway. MK2206 significantly decreased the expression of p-Akt, and AZD8055, an efficient mTOR inhibitor, effectively reduced the level of p-mTOR. In addition, the expression of γH2AX was increased, while RAD51 was decreased, indicating that inhibition of the Akt/mTOR pathway was necessary for niraparib-induced DNA damage (Fig. 6c and d). The CCK-8 assay also proved that the suppression of Akt or mTOR led to further inhibition of cell proliferation (Fig. 6b).

### STS enhances the antitumour activity of niraparib in xenograft animal models

**Fig. 7:**
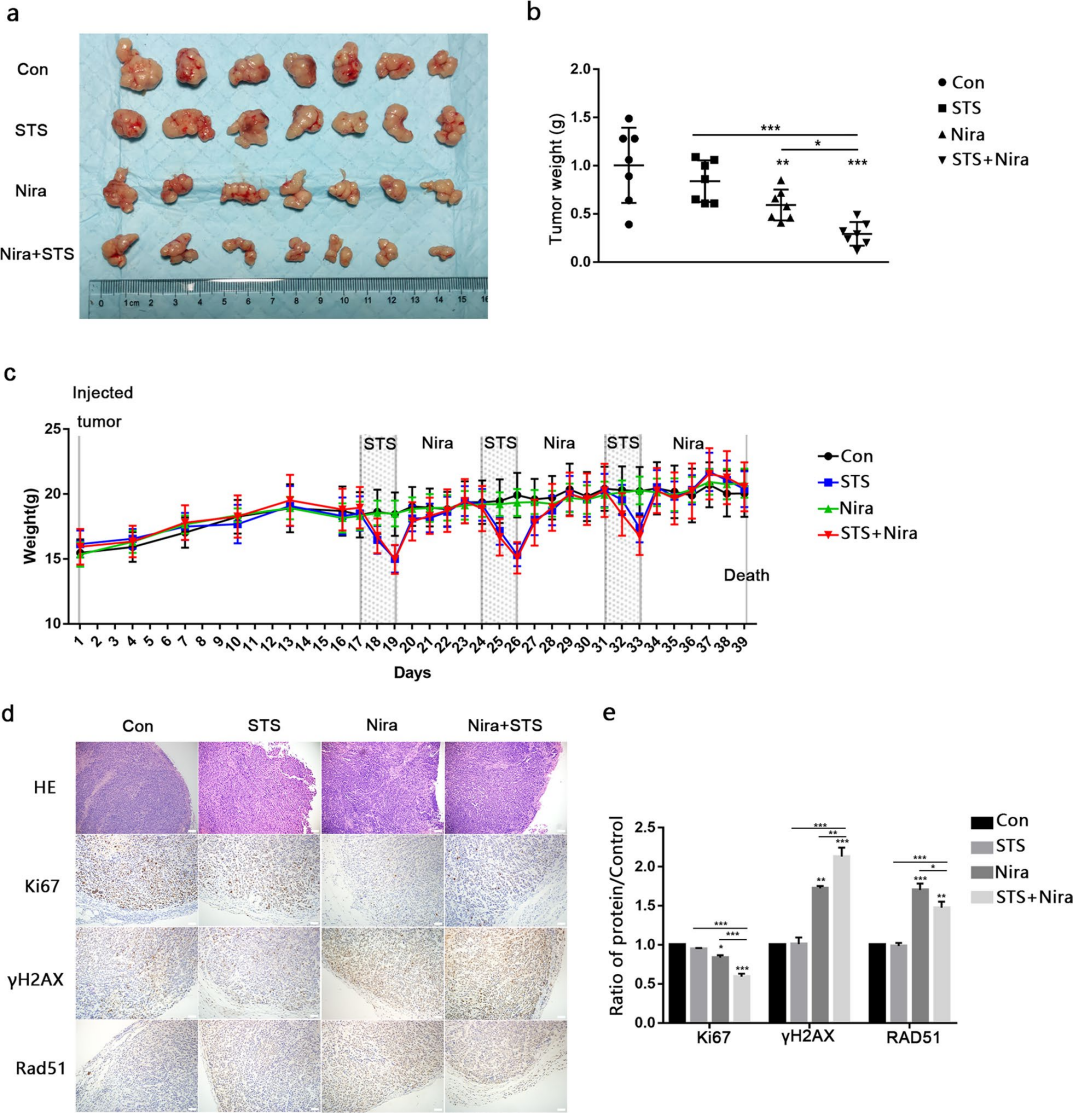
**a** Images of subcutaneous tumours after treatment with niraparib, STS or in combination. **b** The weight of tumours. **c** The weight of the xenograft animals in the control (Con), STS, niraparib (Nira) or STS + niraparib (STS + Nira) groups. **d** HE staining and IHC staining of Ki67, γH2AX and RAD51 were performed, and the statistical results are presented. Scale bar: 200 μm with HE and 100 μm with IHC staining. The values are presented as the mean ± SD (n = 7). ***P < 0.001, **P < 0.01, *P < 0.05

We then generated xenograft tumour models to determine the therapeutic efficacy of STS + niraparib combination therapy. The xenograft mice were randomly divided into 4 groups: control, STS, niraparib (50 mg/kg) and STS + niraparib. As shown in Fig. 7, compared with the control group, the tumour weight in the niraparib group was reduced after treatment, and the reduction in the STS + niraparib group was more obvious (Fig. 7a and b). Our results demonstrated that the combination with STS enhanced the antitumour activities of niraparib. In addition, we also recorded the trend of weight change in this study. The body weight of the mice in the niraparib group did not change significantly, while the body weight of the mice after 48 h starvation decreased by approximately 20%, but the body weight returned to normal 72 h after returning to a normal diet (Fig. 7c). This meant that STS only affected the weight of mice in the short term. HE staining showed a large area of tumour tissue necrosis after niraparib treatment. IHC staining showed that compared with the niraparib group, the expression of Ki67 and RAD51 in the STS + niraparib group decreased, and γH2AX increased, which meant that STS enhanced niraparib-induced cell proliferation arrest and DNA damage. (Fig. 7d and e).

### STS reduces the side effects of niraparib in xenograft animal models

**Fig. 8:**
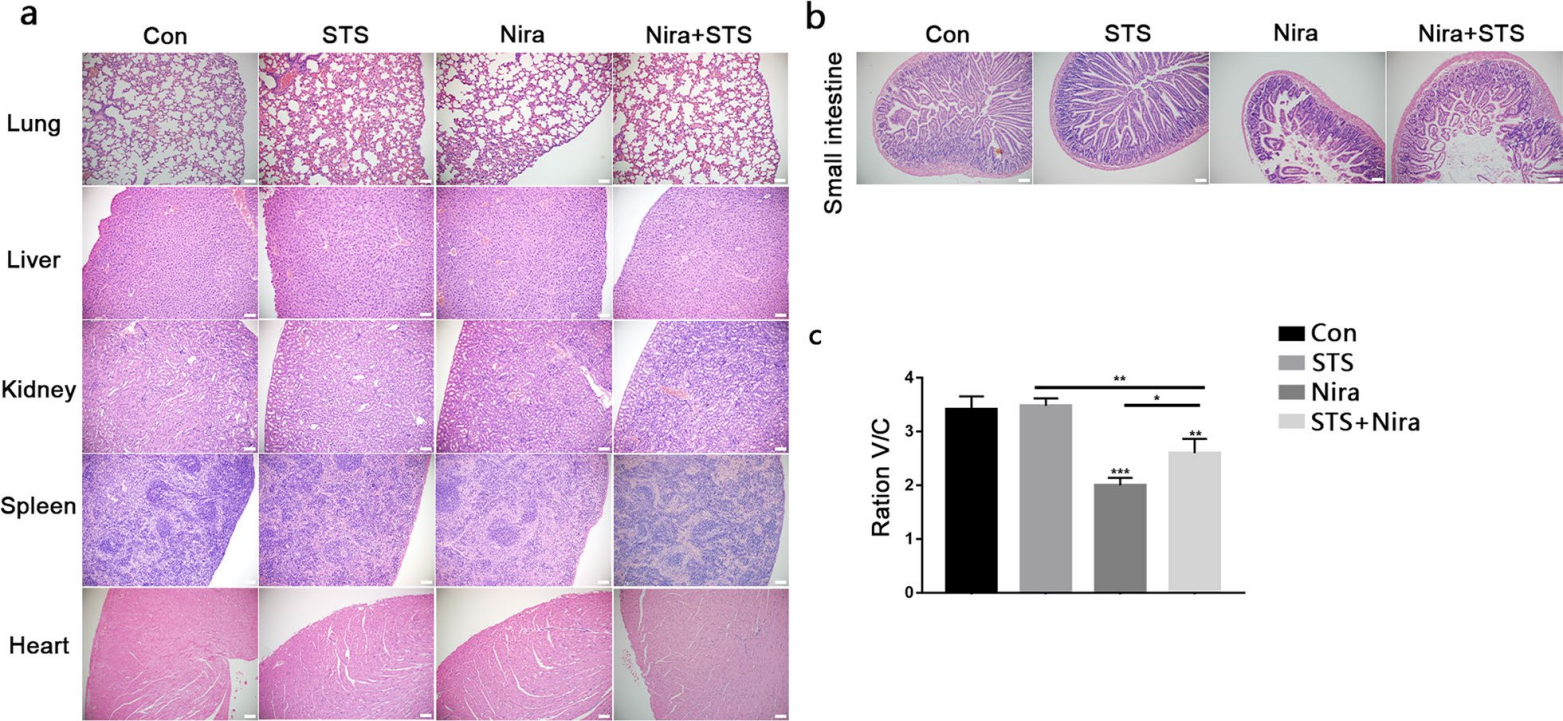
**a** HE staining of different organs (lung, liver, kidney, spleen and heart). **b**, **c** HE staining of small intestine. Data are represented as the mean ± SD of three independent experiments (n = 7). ***P < 0.001, **P < 0.01, *P < 0.05

Side effects cannot be ignored during the process of tumour chemotherapy. We asked whether STS affected the side effects while enhancing the effect of chemotherapy. We performed HE staining of major organs (lung, liver, spleen, heart, kidney), and there was no significant organ-related toxicity in the niraparib and STS + niraparib groups compared with the control (Fig. 8a).

Gastrointestinal toxicity has been reported as a significant adverse effect of niraparib in ENGOT-OV16/NOVA clinical trials [22]. We performed HE staining of the intestinum tenue and calculated the value of V/C (the ratio of the villi length by the intestinum tenue/crypt depth). Our experimental results demonstrated that compared with the control and STS groups, 3 weeks of niraparib treatment resulted in a shorter length and broken small intestinal villi, including less crypt depth, while STS reduced the digestive side effects induced by niraparib to a certain extent (Fig. 8b and c). These data suggested that combination therapy with STS and niraparib enhanced antitumour activity in vivo without distinct organ-related toxicity. In addition, STS can also synergistically reduce the intestinal side effects induced by niraparib.

## Discussion

The value of fasting during cancer therapy has gradually attracted attention. Fasting can prolong the lifespan of organisms ranging from yeast to mice [23, 24]. Compared with long-term calorie restriction, STS is easier to implement and complete because of less weight loss and better enforceability. The efficacy of STS combined with chemotherapy or radiotherapy in the treatment of malignant tumours has been confirmed, such as neurocytoma [16], malignant multiforme [24, 25], breast cancer [26] and lung carcinoma [27]. A study has shown that in prostate cancer and pancreatic cancer, STS increases the radiosensitivity of metastatic tumour cells but not normal fibroblasts [28]. In several clinical studies of gynaecological malignancies, STS in combination with chemotherapy is feasible and well tolerated without significant side effects [29, 30]. STS for 48 or 60 h did not result in a significant decrease in body mass, and QOL scores during chemotherapy were improved. In this study, we confirmed that STS enhances the effectiveness of niraparib in ovarian cancer chemotherapy.

Our study showed that STS increased the sensitivity of SKOV3 and A2780 cells to PARPi niraparib treatment. In our experiments, niraparib induced cytotoxicity in SKOV3 and A2780 cells in a time- and dose-dependent manner. Although STS had no effect on the malignant behaviour of ovarian cancer cells, it enhanced the inhibition of cell proliferation and apoptosis induced by niraparib chemotherapy. In addition, consistent with previous research, niraparib induced G2/M phase arrest [31]. A comet assay was used to detect the degree of DNA lesions, including single- and double-strand breaks [32]. Compared with the niraparib group, higher alkali-labile sites and higher tail moments illustrated the efficacy of the combination of STS and niraparib in the treatment of ovarian cancer. In addition, western blot and immunofluorescence analyses demonstrated that compared with the niraparib group, STS pretreatment resulted in an increase in γH2AX and a decrease in RAD51, which meant that high levels of DNA damage and inhibition of HR repair and overloading stress make tumour cells more sensitive to chemotherapy by exhausting the stress response pathway [33].

Mechanistically, overactive PARP induces a derangement of ATP production, accounting for the overconsumption of β-nicotinamide adenine dinucleotide (NAD^+^), resulting in necrosis and autophagy [34, 35]. In our study, autophagic vacuoles were observed by confocal microscopy, and the expression of LC3II and p62 was quantified by western blot analysis. Consistent with other research, niraparib induced enhanced autophagy and high expression of LC3II [36], and the mechanisms of autophagy may be triggered by cellular stress, such as ROS, or genomic instability rather than protective effects. The Akt/mTOR signalling pathway regulates cell growth, proliferation, motility, metabolism and cell size, and inhibition of this pathway promotes tumour regression [37, 38]. Previous studies have shown the main mechanism by which STS enhances the effect of chemotherapy is by inhibiting IGF1/Akt/mTOR signalling [21, 39, 40], and the coordinated repression of p-Akt and m-TOR may be the main reason for the enhanced effect of niraparib. Enhanced autophagy flux and inhibition of the AKT/mTOR signalling pathway were observed after STS and niraparib were used in combination, indicating the important role of AKT/mTOR in this process. Moreover, MK2206 and AZD8055 were applied on the basis of STS and niraparib combination treatment. Our results revealed that inhibition of AKT/mTOR induced stronger cell proliferation inhibition and higher expression of the DNA damage-related protein γH2AX, while the reduction in RAD51 was consistent with impairment of the HR repair mechanism.

Furthermore, we performed in vivo experiments to detect the effect of STS and niraparib in combination in a xenograft nude mouse model. No mice died during the experiment, and STS alone showed no significant effect on tumour growth compared to the control. We found that STS significantly enhanced the antitumour effect of niraparib, and the combination therapy did not show significant major organ-related toxicity (lung, liver, spleen, heart, and kidney). In addition, although STS alone or in combination with niraparib for 48 h caused a sharp drop in the weight of mice, when their normal diet was restored, the weight of the mice returned to normal, which indicated that STS had no effect on weight in the long term. Gastrointestinal toxicity is one of the side effects of most chemotherapeutics, including niraparib. In this study, we observed the protective effect of STS on the small intestine after niraparib application.

In conclusion, our research showed that STS synergistically enhanced the toxicity of niraparib on ovarian cancer cells through the Akt/mTOR signalling pathway. These findings reveal that the combined application of STS and niraparib is a potential maintenance treatment strategy for ovarian cancer. However, our experimental study on the synergistic effect of STS and niraparib was carried out in vitro and at the animal level. In the future, it is necessary to further explore the effect of this combination therapy in clinical research.

## Data Availability

The datasets used and/or analyzed during the current study are available from the corresponding author on reasonable request.
