# Correction to: Short‑term starvation synergistically enhances cytotoxicity of Niraparib via Akt/mTOR signaling pathway in ovarian cancer therapy

**DOI:** 10.1186/s12935-022-02548-4

**Published:** 2022-03-21

**Authors:** Zhi Wang, Suting Li, Yuting Wan, Fuwen Wu, Li Hong

**Affiliations:** Department of Gynecology and Obstetrics, Renmin Hospital of Wuhan University, 238 Jiefang Road, Wuhan, Hubei Province People’s Republic of China

## Correction to: Cancer Cell International 22:18 (2022) https://doi.org/10.1186/s12935-022-02447-8

In the original version of this article [1], the given and family name of Zhi Wang was incorrectly structured. The name was displayed correctly in this correction.
